# Validation of the parent version of the inventory of school attendance problems (ISAP-P)

**DOI:** 10.3389/frcha.2025.1543527

**Published:** 2025-02-25

**Authors:** Martin Knollmann, Volker Reissner

**Affiliations:** LVR Clinic Duesseldorf, Department for Child and Adolescent Psychiatry, Psychosomatics, and Psychotherapy, Clinics of the Heinrich-Heine-University, Duesseldorf, Germany

**Keywords:** school absenteeism, school refusal, truancy, assessment, questionnaire

## Abstract

This study presents the validation of the parent version of the Inventory of School Attendance Problems (ISAP-P), which assesses a broad spectrum of symptoms associated with school attendance problems. A model with 49 items loading on 13 factors derived from the child version showed an acceptable fit (*N* = 296). Correlations with other measures indicated convergent and discriminant validity of the scales, but associations with the extent of school absences were not detected. Concordant scales of the child vs. parent version were correlated in the expected directions, but some scales showed low interrater agreement. Albeit these initial results support the validity of the ISAP parent version, further studies on its psychometric properties as well as on children's and parents diverging views on SAPs are needed.

## Introduction

Students who are absent from school to a problematic degree have been found to have an elevated risk for mental illnesses, unemployment, drug misuse, school dropout, and many other psychosocial problems across the lifespan (1–3). School attendance problems (SAPs) can occur in many different forms. Recent conceptualizations (4) distinguish between school exclusion (school absences initiated by the school, e.g., because of misbehavior), parental withdrawal (parent motivated absences, e.g., keeping the child at home in order to help there), truancy (child-motivated, generally unexcused school absence due to a lack of motivation and without knowledge of the parents, often accompanied by externalizing symptoms) and school refusal (child-motivated, generally excused school absence with knowledge of the parents because of internalizing symptoms such as anxiety, psychosomatic complaints, or depression).

While consensus regarding these definitions has fostered research into SAPs in the recent years, most researchers acknowledge that these constructs can overlap, coexist, or influence one another. E.g., initial truancy can develop into school refusal, and school absences due to depressive symptoms such as loss of energy and interest can be misinterpreted as a part of truant behavior or a conduct disorder (4, 5). Without a thorough and differentiated assessment of the heterogeneous nature of SAPs, interventions cannot be tailored according to the individual needs of school absent youths. Therefore, several instruments with different foci have been developed to obtain a more detailed picture of SAPs [see (6), for a comprehensive review]. The School Non-Attendance Checklist [SNACK (4);] is a short screening tool which can be used to categorize SAPs according to their reasons (parent report) and is the only measure that not only assesses child-motivated SAPs (truancy, school refusal), but also includes items for school exclusion and parental withdrawal. The majority of the available questionnaires, though, focusses on child-motivated SAPs. Instruments like the widely used SRAS-R [School Refusal Assessment Scale Revised; (7, 8)] or the ARSNA [Assessing Reasons for School Non-Attendance; (9)] subsume different aspects of SAPs under rather broad categories or dimensions (e.g., avoidance of negative affect, escape from aversive social situations, pursuit of attention, and pursuit of tangible reinforcement as functions of SAPs in the SRAS-R). The SCREEN [School Refusal Evaluation Scale (10)] and the SEQ-SS [Self-efficacy Questionnaire for School Situations (11)], on the other hand, can be used for an in-depth assessment of selected features of SAPs (e.g., SCREEN-subscale “Anxious Anticipation” as a facet of school refusal).

The Inventory of School Attendance Problems [ISAP, (12)] complements and extends the range of the existing measures by offering both a comprehensive and differentiated screening of SAPs. It incorporates the most relevant aspects of SAPs as separate scales, which assess specific symptoms often associated with SAPs such as depression, social phobia, separation anxiety, and somatic complaints as well as the impact of common stressors associated with SAPs (e.g., bullying, intrafamily problems; see Table 1). Its items were constructed inductively based on answers of patients of a specialized outpatient unit for SAPs during clinical exploration. It offers an integrative assessment of both the presence of a given symptom prior to or at school and its impact on school attendance. In the left column, under the heading “Before or in school/school time…”, the items are presented (e.g., “…I feel sad.”). In the middle column, students first rate how good an item applies to them (heading “Applies to me”). Then, in the right column, students rate how strongly this item is connected to their SAPs (heading “That's why I miss school/attending school is hard for me”; response scale for both questions: never—sometimes—often—most of the time). Separate scores for symptom presence and symptom impact can be calculated for each scale, which enables practitioners to rank symptoms according to their impact on the student's SAPs and thus to identify the most pivotal targets for interventions. For the statistical analyses, however, testlets were formed for each item by aggregating the values of both response scales (13).

**Table 1:** Scales of the inventory of school attendance problems (ISAP, child version).

Scale (Number of Items)	*α*	Item Examples: “Before or in school…”
Depression (6)	.86	“…I am sad.”; “…I feel tired or without energy.”
Social Anxiety (5)	.86	“…I am afraid to say something when other students are around.”
Performance Anxiety (3)	.87	“…I am afraid of exams.”; “…I worry about my school grades.”
Separation Anxiety (4)	.85	“…I worry that something terrible might happen to my parents.”
Agoraphobia/Panic (4)	.75	“…I am afraid of not being able to leave the classroom when I feel bad.”
Somatic Complaints (3)	.82	“…I have pain (e.g., stomachache, headache, …)”; “…I feel sick.”
School Aversion/Attractive Alternatives (4)	.81	“…I want to do something outside rather than being in school.”; “…I just don't feel like going to school or attending specific courses.”
Aggression (3)	.88	“…I get aggressive easily.”
Problems with Peers (4)	.83	“…I feel excluded by my classmates.”; “…I am afraid of being teased or bullied by other students.”
Problems with Teachers (3)	.81	“…I feel put under pressure by one or more teachers.”
Problems with Parents (3)	.85	“…I feel treated unfairly by my parents.”
Problems within the Family (3)	.88	“…I feel bad because of the problems in my family.
Dislike of Specific School (3)	.85	“…I think that I am in a bad school.”; “…I don't like my school.”

Explorative factor analyses (*N* = 245 patients, item pool: 124 items/testlets) resulted in 48 items loading on 13 factors. These 13 scales assess externalizing and internalizing symptoms as well as emotional distress resulting from stressors in the school and family context (see Table 1). The pattern of the scales' associations with the Youth Self Report (YSR), a German version of the SRAS, and with the extent of school absenteeism supported their construct validity. For example, internalizing symptoms in the YSR and SRAS (e.g., scale “Avoidance of Negative Affect”) were associated with ISAP-scales measuring internalizing symptoms, while the scales aggression and school aversion showed stronger correlations with externalizing symptoms (YSR) and the SRAS-scale “Pursuit of Tangible Rewards”. Furthermore, most of the scales showed weak to moderate positive associations with the extent of school absenteeism.

Up to date only a child version of the ISAP is available, so that the perspective of the parents on their child's SAPs is missing. The multi-informant assessment approach is the gold standard in child psychology (14), and models and studies on SAPs empathize their embeddedness in interacting social contexts (15, 16). Parents are one of the most important proximal factors of SAPs; thus, the aim of this study is to validate the parent version of the ISAP (ISAP-P). As a content analysis of clinical parent interviews about their perception of their child's SAPs during the construction phase of the ISAP-P (see below) yielded the same categories than the child interviews for the youth version (12), we expected

- A confirmation of the 13 factors for the parent version (factorial validity).
- Associations with the CBCL (discriminant validity), the parent version of the SRAS (convergent validity), and the extent of school absenteeism (criterial validity) comparable to those obtained for the ISAP child version with respective child versions of the measures mentioned above.

## Method

### Participants

Approval by the ethics committee of the University of Duisburg-Essen and written informed consent from patients and their parents was obtained prior to data collection. Criteria for inclusion were being a parent with sufficient language and reading skills of a child with SAPs, age ≥ 8, an IQ > 69, and no severe mental illness (e.g., psychosis). Only questionnaires with no missing answers were included.

The final sample consisted of 296 parents of patients of a specialized outpatient unit for children and adolescents with SAPs of the Department of Child and Adolescent Psychiatry, Psychosomatics, and Psychotherapy, University Hospital Essen, University of Duisburg-Essen, Germany. Either a single parent or both parents together filled out the ISAP. (only one questionnaire per child). 56% of their children were male; their mean age was 14.4 years (SD = 3.51, range 8–18). 3.1% reported school absences between 0 and 4 school days during the last 12 school weeks, 18.6% up to 12, 27.7% up to 36, and 10.8% up to 48 school days. 15.2% reported that their child missed more than 48 days and 23.3% stated that their child did not attend school at all (1.3% missing values).

### Measures

#### ISAP-P

The construction of items of the parent version followed the procedure used for the child version. Content analysis of clinical parent interviews prior to item construction (*N* = 162) yielded twenty-five aspects of SAPs identical to those obtained for the child version (12). For each aspect, three to five items were generated, resulting in an item pool of 124 items parallel to those of the child version. Out of these, the 48 items parallel to those of the final ISAP child version were used for the analyses.

#### Other measures

A modified German version of the parent version of the School Refusal Assessment Scale (ESV-P-R) was administered (17). Its three scales measure three symptom clusters of SAPs and their associated functions in terms of positive or negative reinforcement: Separation Anxiety/Attention Seeking Behavior, School Anxiety/Avoidance of Negative Affect, and Alternative Activities/Tangible Rewards. The German version of the Child Behavior Checklist was used to assess general psychopathology [CBCL; (18), scales: Withdrawn, Somatic Complaints, Anxiety and Depression, Social Problems, Thought Problems, Attention Problems, Aggressive Behavior, Delinquent Behavior]. The extent of school absence during the last twelve weeks was measured by a question on the first page of the ISAP-P (0–4 days—all days, see above). For a comparison of the child and the parent version of the ISAP, *N* = 145 ISAP child versions were available.

### Data analysis

In order to test if the factor structure of the ISAP child version can be replicated for the parent version, we used explorative structural equation modelling (19). This approach integrates the advantages of explorative and confirmative factor analysis. It is recommended for testing the structure of measures at a rather early stage of development with high numbers of assumed factors, as it allows integrating cross loadings in the model specification (20). This approach was chosen as the current stage of development of the ISAP-P is somewhere between exploration (parents as different source of information, high probability of necessary changes compared to the child version) and confirmative validation of its structure (approach to data analysis informed by the results of the child version, preselected items, 13 factors expected). The characteristics of the resulting scales and their construct validity were analyzed using descriptive statistics, *T*-tests, and correlations.

## Results and discussion

### Internal structure

Initial analyses of the 48 items parallel to those of the child version showed an inadequate fit of the model. Items with ambiguous loading patterns were deleted or tentatively replaced by other items from the item pool generated for the same SAP aspect or scale (see Table 2 for details). E.g., the item “…my child feels unhappy.” of the scale “Depression” was replaced by the item “…my child does not like him-/herself.”. Furthermore, one item was added to each of the scales “Problems within the Family” and “Performance Anxiety” because they showed an insufficient differentiation with regard to other scales (“Problems with Parents” and 'Social Phobia', respectively, see Table 2). Following the modification indices, ten covariances between error terms of items from the same scale, with identical item stems, and very similar wording were modelled. These modifications led to an acceptable model fit (CFI = .93; RMSEA = 0.05) and clear loading patterns (see Table 2), while other tested model specifications with less or more factors showed an unacceptable fit (e.g., models with 9, 10,11, or 14 factors: CFI < .85). Despite the small item numbers, for all scales Cronbach's α was ≥.75. As expected, the integration of cross loadings resulted in decreased scale intercorrelations compared to those of the child version (12). While the pattern of most scale intercorrelations mirrored the respective scales' affiliation to the internalizing vs. externalizing spectrum, the scales measuring stressors in the family or school context showed weak to moderate associations with both internalizing and externalizing scales.

**Table 2:** Results of the ESEM-model.

Item	Factor/ISAP-P Scale												
Before or in school…	Social Phobia	Depression	Separation Anxiety	Problems w. Peers	School Aversion	Aggression	Problems w. Parents	Somatic Complaints	Performance Anxiety	Problems w. spec. School	Problems w. Family	Agoraphobia/Panic	Problems w. Teachers
…my child is afraid to say something when other students are around (e.g., during the breaks).	**.40**			.35									
…my child is afraid to speak to other people or that others might speak to me.	.**45**												
…my child is afraid of having to say something in front of the entire class (e.g., giving presentations, being called to respond).	.**44**												
…my child worries that he/she might embarrass him-/herself.	.**40**												
…my child is afraid of being asked about the reasons for his/her former absences from or coming too late to school.	.**84**												
…my child does not like him-/herself.^b^		.**43**											
…my child is sad.		.**87**											
…my child feels down or depressed.		.**71**											
…my child has no hope anymore that his/her situation in school will improve.		.**46**											
…my child feels tired or without energy.		.**58**			.31			.32					
…y child longs for his/her parents and wants to stay with them.			.**82**										
…my child misses his/her parents.			.**79**										
…my child is afraid of being in school for such a long time instead of being with his/her parents.			.**59**										
…my child worries that something terrible might happen to his/her parents.			.**57**										
…my child feels bad because he/she gets into arguments with one or more of his/her classmates.				.**74**									
…my child feels excluded by his/her classmates.				.**97**									
…my child feels unhappy because he/she only has a few friends at school.				.**71**									
…my child is afraid of being teased or bullied by other students.				.**73**									
…my child wants to do something at home that is more fun than school.					.**76**								
…my child wants to do something outside rather than being in school.					.**49**								
…my child just does not feel like going to school or attending specific courses.					.**74**								
…my child thinks that it is OK if he/she skipped school once in a while.					.**82**								
…my child gets aggressive easily.						.**84**							
…my child gets irritable easily.						.**73**							
…my child is easily provokable.						.**86**							
…my child feels rejected by his/her parents.							.**83**						
…my child feels that his/her parents do not care about him/her.							.**80**						
…my child thinks that his/her parents do not like him/her.^a^							.**79**						
…my child has pain (e.g., stomachache, headache, …)								.**87**					
…my child feels sick.								.**84**					
…my child feels sick to his/her stomach, has to throw up, or has diarrhea.								.**65**					
…my child worries about his/her school grades.									.**54**				
…my child is afraid of exams.									.**97**				
…my child worries about doing bad in school.									.**40**				
…my child is afraid of tests.^b^									.**83**				
…my child would prefer to attend another school.^a^										**.99**			
…my child wants to go to another school.^a^										**.96**			
…my child does not like his/her school.										**.53**			
…my child is concerned about problems or incidents in his/her family.											**.86**		
…my child feels bad because of the problems in his/her family.											**.78**		
…my child is worried or sad because he/she cannot handle the problems in his/her family.											**.81**		
…my child is worried or sad because of family issues.^b^											**.84**		
…my child is afraid of vomiting or wetting his/her pants before he/she is able to leave the classroom.												.**72**	
…my child is afraid that he/she will not be able to leave the classroom in time before something embarrassing happens to him/her.	.30											.**66**	
…my child is afraid of not being able to leave the classroom when he/she feels bad.												.**68**	
…my child is afraid about losing control over his/her body.^a^												.**58**	
…my child feels treated unfairly by his/her teachers.^a^													.**94**
…my child does not feel well because of his/her problems with one or more teachers.													.**78**
…my child thinks that one or more teachers are against him/her.													.**93**
Correlations	(1)	(2)	(3)	(4)	(5)	(6)	(7)	(8)	(9)	(10)	(11)	(12)	(13)
Social Phobia (1)													
Depression (2)	.43												
Separation Anxiety (3)	.24	.32											
Problems w. Peers (4)	.27	.38	.17										
School Aversion (5)	-	-	-	-									
Aggression (6)	-	.33	.21	.24	.45								
Problems w. Parents (7)	.24	.35	.19	.23	.36	.46							
Somatic Complaints (8)	-	.43	.19	-	-	-	.19						
Performance Anxiety (9)	.36	.38	.18	-	-	.19	.24	-					
Problems w. spec. School (10)	-	-	-	.39	-	.22	-	-	-				
Problems w. Family (11)	.34	.49	.48	.24	.19	.29	.47	.22	-	-			
Agoraphobia/Panic (12)	.27	.21	.26	-	-	-	-	.33	.16	-	-		
Problems w. Teachers (13)	.24	-	-	.29	.27	.32	.32	-	.26	.36	-	-	

Only loadings ≥ .30 and significant correlations (*p* < .05) are depicted. Loadings ≥.40 are in bold.

Comparison with child version:

^a^ Item exchanged.

^b^ Item added. Items without equivalent in the parent version: “…I feel unhappy.”; “…I think that I will never be able to solve my school problems”.

### Construct validity

The correlations between the ISAP, the CBCL, the ESV-P-R, and the extent of school absences are depicted in Tables 3, 4. Regarding convergent validity, the expected pattern of associations was obtained. E.g., school anxiety as measured by the ESV-P-R correlated strongly with the ISAP-P scales “Social Phobia” and “Peer Problems”, the ESV-P-R scale “Alternative Activities” was associated with the ISAP-P scales “School Aversion”, and the separation anxiety scales of both measures were highly correlated. ISAP-P scales measuring internalizing symptoms such as depression and anxiety showed marked correlations with the respective CBCL scales, while the ISAP-P scales “School Aversion” and “Aggression” showed strong associations with the CBCL scales measuring externalizing symptoms. The discriminant validity was supported by the magnitude of these associations, which was only moderate (vs. high). Furthermore, for the ISAP-P scales measuring stressors in the family and school context, which have no equivalent in the CBCL, the weakest associations were observed.

**Table 3:** Correlations of the ISAP-P scales with scales of the CBCL.

ISAP Scales	CBCL Social Withdrawal	CBCL Somatic Complaints	CBCL Anxious-Depressed	CBCL Social Problems	CBCL Attention Deficit	CBCL Dissocial Behavior	CBCL Aggressive Behavior	CBCL Inter-nalizing	CBCL Exter-nalizing	CBCL Total Score
ISAP Depression	.46**	.26**	.47**	.17*	.12**	.19**	.15*	.51**	.18**	.40**
ISAP Problems with Parents	.27**	.16*	.32**	.15*	.24**	.39**	.39**	.33**	.39**	.37**
ISAP Aggression	.15*		.25**	.17*	.25**	.56**	.62**	.23**	.62**	.46**
ISAP Problems with specific School			.15*	.21**		.27**	.23**		.24**	.19**
ISAP Separation Anxiety		.17*	.24**	.23**				.22**	.16*	.23**
ISAP Agoraphobia Panic	.23**	.27**	.31**	.19**				.35**		.23**
ISAP Problems with Teachers			.15*	.14*	.18*	.26**	.20**	.15*	.23**	.23**
ISAP Social Phobia	.44**		.40**	.36**	.17*			.40**		.28**
ISAP School Aversion	.16*				.23**	.49**	.36**		.43**	.30**
ISAP Somatic Complaints	.34**	.75**	.35**	.16*	.17*			.57**		.34**
ISAP Performance Anxiety	.24**		.33**	.20**	.21**			.31**		.26**
ISAP Problems with Peers	.25**		.52**	.52**	.17*	.18**	.23**	.34**	.23**	.34**
ISAP Problems within the Family	.25**	.18*	.31**	.19**		.15*		.31**	.15*	.19**

CBCL, Child Behavior Checklist; German Version (18).

** *p* < .001.

* *p* < .05.

**Table 4:** Correlations of the ISAP-P scales with the scales of the ESV-P-R.

ISAP Scales	ESV-P-R School Anxiety	ESV-P-R Separation Anxiety	ESV-P-R Alternative Activities
ISAP Problems within the Family	.22**	.29**	
ISAP Problems with Peers	.59**	.16*	
ISAP Performance Anxiety	.32**		
ISAP Somatic Complaints	.29**	.22**	−.20**
ISAP School Aversion	−.16*		.57**
ISAP Social Phobia	.62**	.17*	
ISAP Problems with Teachers	.29**		.25**
ISAP Agoraphobia Panic	.43**	.25**	
ISAP Separation Anxiety	.24**	.68**	
ISAP Problems with specific School	.23**		.26**
ISAP Aggression			.39**
ISAP Problems with Parents	.18**		.19**
ISAP Depression	.36**		

ESV-P-R, German version of the parent version of the School Refusal Assessment Scale.

* *p* < .05.

** *p* < .001.

In contrast to the ISAP child version and to findings regarding the scales of the parent version of the SRAS (8), none of the ISAP-P scales showed significant associations with the extent of school absenteeism. Since a parent rating of school absences has been used in this study, these findings could be attributable to a lack of parental knowledge about the real extent of school absenteeism of their child (21). Furthermore, other parental factors that were not assessed and that are known to be associated with school absences (e.g., socioeconomic status) could mediate or moderate the association between the ISAP-P and school attendance rates (22).

The analysis of the interrater agreement in terms of associations between parallel scales of the parent vs. child version of the ISAP yielded mostly moderate to strong correlations (see Table 5). However, for some scales (e.g., Problems within the Family, Performance Anxiety), only weak to moderate correlations were obtained. Comparisons of the means of concordant parent vs. child scales showed significantly higher means of the scales of the parent version in all cases (*T* values between 4.49 and 14.31, all *p* < .0001). This could reflect parents' high levels of worries and distress due to their child's SAPs, possible dissimulation of some children, or conflicts between parent and child (23).

**Table 5:** Correlations between ISAP-P and ISAP child version (*N* = 145).

Child Version Parent Version	Depression	Problems w. Parents	Aggression	Problems w. spec. School	Separation Anxiety	Agoraphobia/Panic	Problems w. Teachers	Social Phobia	School Aversion	Somatic Complaints	Performance Anxiety	Problems w. Peers	Problems w. Family
Depression	.42**		.21*	.24**			.20*	.22**		.26**	.29**	.22**	.21*
Problems w. Parents	.24**	.42**	.30**	.20*					.27**				
Aggression	.26**	.37**	.51**						.26**				.19*
Problems w. spec. School				.67**			.21*		.21*				
Separation Anxiety					.50**	.18*							
Agoraphobia/Panic					.19*	.40**		.17*	−.18*	.23**			
Problems w. Teachers			.25**	.37**			.42**		.27**				
Social Phobia	.29**					.17*	.19*	.38**			.26**	.25**	
School Aversion		.22**	.24**	.18*					.34**				
Somatic Complaints	.21*					.30**				.54**		.18*	
Performance Anxiety							.25**	.18*			.28**		
Problems w. Peers	.25**			.34**				.17*			.19*	.57**	
Problems w. Family		.20*	.21*										.27**

* *p* < .05.

** *p* < .001.

Questions regarding the differences between child and parent version remain to be answered by future research, which should also address the major limitations of this study. Besides the low validity of the measure of school absences mentioned above, important information about the sample is missing (e.g., diagnoses, socioeconomic status), and due to its clinical nature (predominantly youths with severe SAPs) conclusions regarding the factor structure and the construct validity of the ISAP in the general student population cannot be drawn. The factor structure of the child version, however, has recently been confirmed in community samples (24, 25).

Although preliminary, the first results of the evaluation of the ISAP-P indicate that it is a reliable and valid tool for assessing the parent perspective on students' SAPs. Integrating the parents' view in the diagnostic process is essential. Possible “blind spots” or biases of the children's reports can be detected (and vice versa); furthermore, the parent's reports can shed light on their subjective theories about the nature of the problems of their child. Discrepancies between the child's and the parents' conceptualization of the SAP can be detected and integrated into a shared model, which is the prerequisite for the establishment of a treatment plan that both child and parents can agree on (26). Furthermore, results of the ISAP-P can inform psychoeducation and sensitization of parents with regard to their child's problems as well as the design and implementation of parent- or family-centered treatment modules for SAPs. Future studies should cross-validate the ISAP-P in community or school samples and across different cultural contexts. Beyond that, longitudinal studies could use the ISAP and the ISAP-P to investigate the development of SAPs throughout childhood and adolescence from different perspectives, whereas interventional studies could address the potential of the ISAP and the ISAP-P to inform the development of multimodal treatments for SAPs and to evaluate their effectiveness.

## Data Availability

The datasets presented in this article are not readily available. Requests to access the datasets should be directed to martin.knollmann2@lvr.de.
